# Extensive dysautonomia in early-stage multiple system atrophy reflects survival differences: insights from data-driven subtypes

**DOI:** 10.1007/s00415-026-13948-1

**Published:** 2026-06-19

**Authors:** Su Hyeon Ha, Hui-Jun Yang, Seungmin Lee, Kyung Ah Woo, Jung Hwan Shin, Han-Joon Kim

**Affiliations:** 1 Department of Neurology, Seoul National University Hospital, Seoul National University College of Medicine, 101 Daehak-Ro, Jongno-Gu, Seoul, 03080 Republic of Korea; 2 Department of Neurology, Gachon University Gil Hospital, Incheon, Republic of Korea

**Keywords:** Multiple system atrophy, Dysautonomia, Survival analysis, Prognosis, Latent class analysis

## Abstract

**Introduction:**

Multiple system atrophy (MSA) is a clinically heterogeneous disorder. Conventional motor phenotype-based classification provides limited prognostic information. Previously, we identified data-driven subtypes of early-stage MSA using a latent class analysis (LCA) that incorporated both motor and non-motor features. However, the prognostic significance of these subtypes remains unclear.

**Methods:**

We analyzed the survival outcomes in a previously reported cohort of 61 patients with probable or possible MSA enrolled within three years of motor symptom onset. Patients were classified according to LCA-derived subtypes and dichotomized into an extensive or restricted dysautonomia group based on shared autonomic profiles. Overall survival was assessed using Kaplan–Meier analysis and compared using the log-rank test. Cox proportional hazards models were used to estimate hazard ratios (HRs) with progressive adjustments for the age at onset, sex, baseline disease severity (Unified Multiple System Atrophy Rating Scale, UMSARS Part I), and disease duration at enrollment.

**Results:**

Survival analysis was performed in 60 patients, 47 of whom died by the end of follow-up. The extensive dysautonomia group showed a significantly shorter median survival than the restricted group (6.0 vs. 7.0 years; log-rank *p* = 0.008). After adjusting for the age at onset and sex, the restricted group had a lower risk of mortality (adjusted HR: 0.538, 95% CI 0.290–0.996; *p* = 0.049). This association was attenuated after additional adjustments for the baseline disease severity (UMSARS Part I) and disease duration.

**Conclusions:**

Data-driven subtypes defined by early symptom patterns correspond to clinically meaningful survival differences in patients with MSA. Extensive dysautonomia reflects a more malignant, globally severe phenotype than isolated autonomic involvement, highlighting the prognostic relevance of incorporating non-motor features into early MSA classification.

**Supplementary Information:**

The online version contains supplementary material available at 10.1007/s00415-026-13948-1.

## Introduction

Multiple system atrophy (MSA) is a highly heterogeneous disorder, and conventional classification based on dominant motor symptoms—prominent parkinsonism (MSA-P) and prominent cerebellar ataxia (MSA-C)—does not provide a comprehensive understanding of patient prognosis [1]. Previously, we conducted a latent class analysis (LCA) of patients with MSA within three years of motor symptom onset, assigned even weights to both motor and non-motor features, and identified three distinct subtypes: class 1 (moderate parkinsonism + extensive dysautonomia), class 2 (predominant parkinsonism + limited dysautonomia), and class 3 (predominant cerebellar symptoms + limited dysautonomia) [2]. These descriptors do not indicate absolute severity but rather reflect the relative prominence of clinical features within each class. Class 1 patients exhibited extensive autonomic dysfunction, whereas patients in classes 2 and 3 shared the characteristics of limited dysautonomia [2]. Although LCA identified cross-sectional heterogeneity, the prognostic significance of these subtypes—derived independently of outcome information—remains unclear. Therefore, this study aims to investigate whether these data-driven subtypes of early-stage MSA are associated with differential survival outcomes.

## Methods

We analyzed a cohort of patients with early-stage MSA originally reported in our previous study, using LCA [2]. Briefly, 61 patients with probable or possible MSA with a disease duration of ≤ 3 years at baseline were prospectively enrolled at Seoul National University Hospital between June and December 2018. The diagnosis was made according to the second consensus criteria [3], and all patients were followed up for at least two years to confirm diagnostic stability. For the present study, survival outcomes were assessed with follow-up data available until June 30, 2025, including the mortality status and time from symptom onset to death or censoring.

Patients were classified according to the LCA-derived subtypes described previously [2], where the extensive dysautonomia subtype (class 1) was characterized by a high probability of autonomic symptoms, particularly orthostatic symptoms and bowel dysfunction. Clinical and imaging variables in the original LCA dataset were evaluated according to standardized procedures. Autonomic symptoms were assessed using the Unified Multiple System Atrophy Rating Scale (UMSARS) Part I items—orthostatic symptoms (item 9), sexual dysfunction (item 11), and bowel dysfunction (item 12). Other non-motor features, including hyperreflexia, emotional incontinence, camptocormia, stridor, snoring, and cold hand, were assessed based on clinical evaluation and recorded as binary variables (present/absent) without formal severity scoring. Probable rapid eye movement sleep behavior disorder (RBD) was assessed using the RBD Single-Question Screen, and putaminal atrophy on brain magnetic resonance imaging (MRI) was treated as a categorical variable (present/absent) based on qualitative radiological assessments performed by independent radiologists. These clinical and imaging variables were not re-evaluated in the present study; instead, previously recorded assessments were used without modifications.

For survival analysis, we grouped the patients into either extensive dysautonomia group (class 1) or restricted dysautonomia group (classes 2 and 3). Classes 2 and 3 were merged owing to their shared pattern of limited autonomic involvement in the original LCA and the small sample size of class 2 (*n* = 8), which limited the statistical power of separate analysis. Baseline demographic, clinical, and imaging characteristics were compared between the two groups using independent-samples t tests or Mann–Whitney U tests for continuous variables and chi-square or Fisher’s exact tests for categorical variables as appropriate. These comparisons were unadjusted and performed using the dichotomized grouping in the present study. Kaplan–Meier survival curves and log-rank tests were used to compare survival distributions. Cox proportional hazards regression models were fitted to estimate the hazard ratios (HRs) and 95% confidence intervals (CIs). To test the robustness of the findings, these analyses were progressively adjusted for potential confounders: age at onset, sex, baseline disease severity (UMSARS Part I score), and disease duration at enrollment. The proportional hazard assumption was evaluated using Schoenfeld residuals, and no violations were detected. The log-linearity of the continuous covariates (age at onset, UMSARS Part I score, and disease duration at enrollment) was assessed using component plus residual plots, which demonstrated acceptable linearity. Statistical significance was set at two-sided *p* values < 0.05. Analysis was performed using SPSS version 30 and R version 4.5.0. The study protocol was approved by the Institutional Review Board of the Seoul National University Hospital.

## Results

A total of 61 patients with MSA in the extensive dysautonomia (*n* = 32) and restricted dysautonomia (*n* = 29) groups were analyzed. Baseline demographic and clinical characteristics are presented in Table 1. Patients in the extensive group were older at onset (60.8 ± 7.2 vs. 56.7 ± 8.8 years; *p* = 0.049) and had significantly higher UMSARS Part I scores (28.9 ± 6.1 vs. 19.4 ± 7.1; *p* < 0.001). The distribution of empirical motor subtypes also differed; MSA-P was predominant in the extensive group, whereas MSA-C was more frequent in the restricted group (*p* = 0.002). Putaminal atrophy observed on MRI was more common in the extensive group (46.7% vs. 11.1%; *p* = 0.003). Group differences in autonomic symptoms are detailed in Supplementary Table S1.

**Table 1:** Demographic and clinical characteristics of patients with multiple system atrophy: extensive versus restricted dysautonomiaThis cohort was previously reported by Yang et al. [2]; baseline characteristics were reanalyzed using a dichotomized grouping for the present survival analysis

Characteristics	Extensive: Moderate parkinsonism + extensive dysautonomia	Restricted: Predominant motor symptoms + limited dysautonomia	*χ*^2^	*P*	
Number of patients, number (%)	32 (52.5%)	29 (47.5%)	–	–	
Onset age, years	60.84 (7.23)	56.72 (8.79)	2.006^a^	0.049	*
Motor symptom duration, years	2.44 (0.56)	2.24 (0.51)	376.5^b^	0.142	
Sex, male, number (%)	17 (53.1%)	19 (65.5%)	0.966^c^	0.326	
Empirical motor subtype			12.213^c^	0.002	*
MSA-P, number (%)	17 (53.1%)	5 (17.2%)			
MSA-C, number (%)	10 (31.3%)	22 (75.9%)			
MSA-PC, number (%)	5 (15.6%)	2 (6.9%)			
Hoehn and Yahr stage	4.16 (0.72)	3.66 (0.81)	306.5^b^	0.106	
UMSARS part I score	28.91 (6.07)	19.38 (7.10)	5.648^a^	< 0.001	*
MRI findings (*n* = 57)					
Putaminal atrophy, number (%)	14 (46.7%)	3 (11.1%)	8.584^c^	0.003	*
Cerebellar atrophy, number (%)	27 (90.0%)	22 (81.5%)	0.855^c^	0.355	
Number of deaths, number (%)	28 (90.3%)	19 (65.5%)	5.432^b^	0.020	*
Median survival time, years (IQR)	6.0 (5.0–8.0)	7.0 (6.0–9.0)	7.127^d^	0.008	*

Data are presented as means (standard deviation) unless otherwise indicated; median values are shown with interquartile ranges

For the empirical motor subtype, some cells had expected counts < 5; therefore, the Fisher–Freeman–Halton exact test with Monte Carlo simulation (10,000 samples, 95% CI) was additionally conducted, which confirmed the significance (*p* = 0.003, 95% CI 0.002–0.004)

^a^ Independent-samples *t* test

^b^ Mann–Whitney *U* test

^c^ Chi-square test

^d^ Log-rank test

^*^ *p* < 0.05

Percentages were calculated based on available data, excluding missing values

**Fig. 1:**
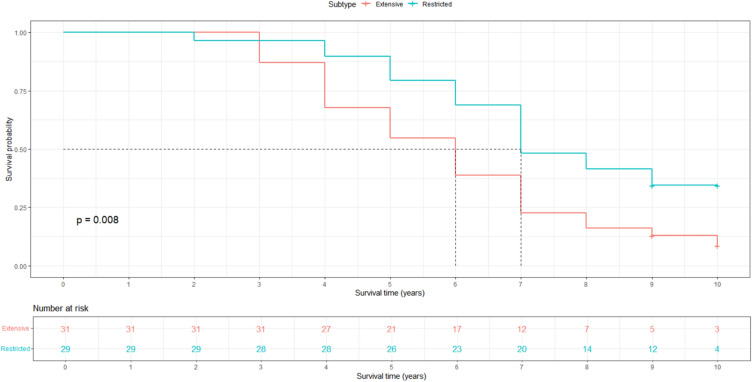
Kaplan–Meier survival curves of patients with multiple system atrophy with extensive versus restricted dysautonomia group

Mortality status could not be confirmed in one patient. Therefore, survival analysis was performed in 60 patients, among whom 47 (78.3%) had died as of June 30, 2025, when the mortality status was assessed. The median survival time of the 60 patients was 7.0 years (95% CI 6.2–7.8). The mortality rate was significantly higher in the extensive group than in the restricted group (90.3% vs. 65.5%; *p* = 0.020). The median survival time from motor onset was shorter in the extensive group (6.0 years, 95% CI 4.8–7.2) than in the restricted group (7.0 years, 95% CI 5.7–8.3; log-rank *p* = 0.008) (Fig. 1).

Kaplan–Meier survival curves illustrating overall survival probability for patients with multiple system atrophy with extensive versus restricted dysautonomia group. Median survival times are indicated with horizontal and vertical dashed lines. The accompanying risk table displays the number of patients at risk at each time point. Log-rank test: *p* = 0.008. Adjusted hazard ratio for the restricted group relative to the extensive group, controlling for age at onset and sex: HR = 0.538 (95% CI 0.290–0.996).

In the Cox regression analysis (Table 2), the restricted group showed a lower risk of mortality than the extensive group after adjusting for the age at onset and sex (adjusted HR: 0.538, 95% CI 0.290–0.996; *p* = 0.049). After additional adjustment for the baseline disease severity (UMSARS Part I score), the association was attenuated (adjusted HR: 0.857, 95% CI 0.408–1.801; *p* = 0.683), suggesting that functional impairment at enrollment partly mediated the relationship between the dysautonomia burden and survival. In a sensitivity analysis that further included the disease duration at enrollment (**Supplementary Table S2**), both the disease duration and the baseline severity emerged as independent predictors of mortality, whereas the subgroup effect was no longer significant (HR: 0.682, 95% CI 0.322–1.447; *p* = 0.319), indicating that these clinical factors largely accounted for the survival differences between subgroups. Model diagnostics showed no violation of the proportional hazards assumption (all Schoenfeld residual tests, *p* > 0.05; global, *p* = 0.88), and the component plus residual plots demonstrated acceptable log-linearity for continuous covariates.

**Table 2:** Hazard ratios for mortality in patients with multiple system atrophy with extensive versus restricted dysautonomia group

Group	Crude HR (95% CI)	*p* value	Adjusted HR* (95% CI)	*p* value	Adjusted HR** (95% CI)	*p* value
Extensive (reference)	1.00		1.00		1.00	
Restricted	0.490 (0.272–0.881)	0.017	0.538 (0.290–0.996)	0.049	0.857 (0.408–1.801)	0.683

^*^Adjusted for age at onset and sex

^**^Adjusted for age at onset, sex, and UMSARS Part I score

*HR* hazard ratio, *CI* confidence interval

## Discussion

A previous study using LCA that evenly weighted motor and non-motor symptoms identified three subtypes—class 1 (moderate parkinsonism + extensive dysautonomia), class 2 (predominant parkinsonism + limited dysautonomia), and class 3 (predominant cerebellar symptoms + limited dysautonomia)—and suggested that these subtypes may represent distinct clinical phenotypes [2]. By incorporating longitudinal survival outcomes, this study extends the earlier work by evaluating whether these data-driven subtypes—defined independently of outcome information—are associated with differential survival. Our findings suggest that prognostic differences in MSA may be better understood in terms of early clinical patterns rather than isolated features. Patients in the extensive dysautonomia group showed a broader pattern of multisystem involvement at baseline, which was associated with poorer survival. However, the attenuation of this association after adjustment indicates that this pattern likely reflects overall disease burden rather than acting as an independent prognostic factor. Notably, Hoehn and Yahr staging did not differ significantly between groups.

Notably, the LCA model was constructed solely from early motor and non-motor features without incorporating prognostic information. The observation that these phenotype-based subgroups also diverged in survival provides supporting evidence that integrating non-motor domains enhances the ability to capture clinically relevant distinctions that extend beyond the cross-sectional presentation. In this context, the poorer outcomes in the extensive dysautonomia group highlight the value of a broader multisystem perspective when characterizing early-stage MSA.

Patients in the extensive dysautonomia group showed higher UMSARS Part I scores, a greater frequency of putaminal atrophy on MRI, and a significantly shorter survival than those in the restricted dysautonomia group. These findings are consistent with those of previous reports that early autonomic failure is a poor prognostic factor for MSA [4–6]. Our previous LCA identified that despite comparable ages at onset and disease durations, the three subgroups exhibited markedly different motor and non-motor symptom profiles, indicating that MSA heterogeneity cannot be explained by simple severity gradation. When these data-driven subtypes were reframed into two broader categories in the present study (extensive vs. restricted dysautonomia), a comparable pattern emerged: patients with extensive dysautonomia showed more widespread clinical involvement and poorer survival. Importantly, the extensive group displayed a greater symptom burden across multiple domains—motor, autonomic, and non-motor—consistent with a more globally severe early phenotype than isolated autonomic involvement.

Notably, the survival difference between the subgroups was attenuated after adjusting for the baseline severity and disease duration. Thus, much of the crude survival gap was explained by differences in the baseline functional status and how long the patients had been living with the disease at the time of enrollment. However, the disease duration at enrollment did not differ significantly between the subgroups, suggesting that the poorer survival of the extensive group cannot be attributed simply to being at a later disease stage. Instead, the extensive subtype appears to reflect a more severe and broadly distributed disease phenotype. Although the extensive group tended to be slightly older at the onset, this modest difference does not fully explain the divergent clinical trajectories observed between the two subtypes. Collectively, these findings reinforce the concept that data-driven subtypes represent distinct clinical phenotypes rather than sequential stages along a single continuum.

To further clarify the role of the disease duration at enrollment in our models, it is important to note that although the disease duration at enrollment and UMSARS part I scores are related to disease progression, they represent complementary clinical dimensions. The UMSARS reflects the degree of functional impairment at the time of the baseline assessment, whereas the disease duration at enrollment reflects how long the disease has been evolving up to that point, an aspect that varies substantially even among patients enrolled within three years of onset. Incorporating the disease duration into the sensitivity analysis allowed us to adjust for differences in the baseline disease stage that were not fully explained by the functional severity and to determine whether the observed subgroup differences in survival persisted after accounting for this variation.

Taken together, the attenuation of subgroup differences after adjustment suggests that the extensive subtype reflects a more malignant and globally severe disease phenotype rather than autonomic dysfunction that acts as an independent causal factor.

Our findings have potential clinical implications. Early identification of patients with a broader pattern of multisystem involvement, particularly those with extensive dysautonomia, may help clinicians recognize individuals at higher risk of rapid disease progression. This phenotype-based perspective may also be useful for patient stratification in clinical trials targeting early-stage MSA, where heterogeneity in disease course can confound treatment effects, while complementing conventional motor-based classifications by capturing additional dimensions of disease heterogeneity.

In terms of pathophysiology, the extensive dysautonomia group may reflect the early and widespread neuropathological involvement of the central autonomic structures, including the brainstem and spinal cord nuclei, whereas the restricted dysautonomia group may indicate more localized or delayed involvement [7, 8]. These mechanistic differences may account for the distinct survival patterns and further support the validity of the distinct phenotypic hypotheses.

This study had several limitations. First, the sample size was relatively small (*n* = 61) and was derived from a single center, which may limit its generalizability. Second, diagnosis was based on clinical criteria without pathological confirmation. The determination of disease duration based on reported symptom onset may be imprecise due to the insidious onset of MSA, potentially introducing variability in the classification of early-stage disease. Third, detailed causes of death were not available; therefore, we could not exclude the possibility that some deaths were unrelated to MSA. Fourth, while the follow-up duration was adequate to estimate the median survival, a longer follow-up is required to capture the full survival spectrum as some patients remain alive. Fifth, classes 2 and 3 were merged into a single restricted dysautonomia group. Although this was clinically reasonable, the very small number of class 2 patients (*n* = 8) limited the statistical power for separate analysis, and any prognostic differences between the two subgroups could not be fully assessed. In addition, although overall motor severity was partly assessed using Hoehn and Yahr staging, the absence of a detailed motor rating scale such as UMSARS Part II limits our ability to fully evaluate differences in motor symptom burden between groups. Furthermore, information on interventions such as tracheostomy or continuous positive airway pressure (CPAP), which may influence survival, was not available and therefore could not be accounted for in the analysis. Finally, internal validation procedures such as bootstrap resampling were not performed because of the modest sample size, and the study was not designed to provide mechanistic insight into the identified phenotypes.

Future studies are needed to validate these findings in larger multicenter cohorts. In addition, incorporating quantitative autonomic function testing along with conventional motor and non-motor assessments may help to better characterize autonomic involvement and further refine the prognostic stratification in early-stage MSA.

In summary, this study showed that patients with the data-driven subtype characterized by early extensive dysautonomia, reflecting broader multisystem involvement, had substantially poorer survival. These findings indicate that prognostics in MSA cannot be fully captured by conventional motor phenotype classification alone. Therefore, incorporating non-motor domains, particularly autonomic dysfunction, into early phenotypic frameworks may provide a more meaningful representation of the underlying disease trajectories and enhance future attempts to stratify patients in the early stages of the disease.

## Supplementary Information

Below is the link to the electronic supplementary material.

Supplementary file1 (DOCX 18 KB)

## Data Availability

Not applicable.
