# Supplementary material for: Extensive dysautonomia in early-stage multiple system atrophy reflects survival differences: insights from data-driven subtypes
Source: J Neurol. 2026 Jun 19;273(7):406. doi: 10.1007/s00415-026-13948-1 (PMC13282191; doi:10.1007/s00415-026-13948-1)
Supplement: Supplementary file 1 — Supplementary file1 (DOCX 18 KB) [file 415_2026_13948_MOESM1_ESM.docx]

**Supplementary Table S1.** Autonomic dysfunction differentiating extensive and restricted dysautonomia

| **Autonomic symptom** | **Extensive** | **Restricted** |
| --- | --- | --- |
| Orthostatic symptom | 87.5% (28/32) | 41.4% (12/29) |
| Bowel dysfunction | 100% (32/32) | 62.1% (18/29) |

Urinary and sexual dysfunction were not included in the original latent class analysis model because they were present in nearly all patients.

**Supplementary Table S2**. Hazard ratios for mortality after additional adjustment for disease duration at enrollment

| **Covariate** | **B** | **SE** | **Wald** | ***p*-value** | **HR (95% CI)** |
| --- | --- | --- | --- | --- | --- |
| Age at onset | 0.011 | 0.021 | 0.294 | 0.587 | 1.011 (0.971-1.053) |
| Sex | 0.297 | 0.319 | 0.866 | 0.352 | 1.346 (0.720-2.518) |
| Disease duration (at enrollment, years) | -0.863 | 0.334 | 6.689 | 0.010 | 0.422 (0.219-0.811) |
| UMSARS part I score | 0.061 | 0.024 | 6.795 | 0.009 | 1.063 (1.015-1.114) |
| Group (Restricted vs. Extensive) | -0.382 | 0.383 | 0.993 | 0.319 | 0.682 (0.322-1.447) |

Cox proportional hazards regression model.

HR, hazard ratio; CI, confidence interval.
